# Time-Resolved Analysis of N-RNA Interactions during RVFV Infection Shows Qualitative and Quantitative Shifts in RNA Encapsidation and Packaging

**DOI:** 10.3390/v13122417

**Published:** 2021-12-02

**Authors:** Miyuki Hayashi, Eric P. Schultz, Jean-Marc Lanchy, J. Stephen Lodmell

**Affiliations:** 1 Department of Chemistry and Biochemistry, University of Montana, Missoula, MT 59812, USA; miyuki.hayashi@umontana.edu; 2 Center for Biomolecular Structure and Dynamics, Missoula, MT 59812, USA; eric.schultz@umontana.edu; 3 Division of Biological Sciences, University of Montana, Missoula, MT 59812, USA; jean-marc.lanchy@umontana.edu

**Keywords:** Rift Valley fever virus, nucleocapsid protein, CLIP-seq, MRM-MS

## Abstract

Rift Valley fever virus (RVFV) is a negative-sense, tripartite RNA virus that is endemic to Africa and the Arabian Peninsula. It can cause severe disease and mortality in humans and domestic livestock and is a concern for its potential to spread more globally. RVFV’s nucleocapsid protein (N) is an RNA-binding protein that is necessary for viral transcription, replication, and the production of nascent viral particles. We have conducted crosslinking, immunoprecipitation, and sequencing (CLIP-seq) to characterize N interactions with host and viral RNAs during infection. In parallel, to precisely measure intracellular N levels, we employed multiple reaction monitoring mass spectrometry (MRM-MS). Our results show that N binds mostly to host RNAs at early stages of infection, yielding nascent virus particles of reduced infectivity. The expression of N plateaus 10 h post-infection, whereas the intracellular viral RNA concentration continues to increase. Moreover, the virions produced later in infection have higher infectivity. Taken together, the detailed examination of these N–RNA interactions provides insight into how the regulated expression of N and viral RNA produces both infectious and incomplete, noninfectious particles.

## 1. Introduction

Rift Valley fever virus (RVFV) is a single-stranded RNA virus that belongs to the order Bunyavirales. RVFV is transmitted by *Aedes* and *Culex* mosquito species and is prevalent in sub-Saharan Africa and the Arabian Peninsula [1]. RVFV infects livestock, causing hepatitis, hemorrhage, abortion, and death [2]. The high abortion rate caused by RVFV and resultant loss of livestock represents a significant threat to local economies [3]. Transmission to humans can occur both through mosquito bites and by contact with infected agricultural animals or animal products [4]. Symptoms of RVFV infection in humans vary from mild flu-like illness to vision loss, liver damage, hemorrhagic fever, and death. Furthermore, recent studies have shown that RVFV infection can be abortive in mice [5], and likely in humans [6,7]. No approved vaccine or treatment is currently available for RVFV infection in humans.

RVFV has a tripartite, single-stranded RNA genome, designated as the small (S), medium (M), and large (L) segments. The S segment encodes the nucleocapsid protein (N) and nonstructural protein S (NSs), which is the major virulence factor that interferes with host transcription and antiviral response [8,9]. The M segment encodes two glycoproteins, Gn and Gc, as well as P78 and nonstructural protein NSm. Gn plays a critical role in packaging of viral RNAs (vRNAs) into virions through interactions with N and the RNA-dependent RNA polymerase (RdRp), and via direct interactions with vRNA [10,11,12]. The L segment encodes the RdRp. The M and L segments are negative-sense, whereas the S segment encodes the N and NSs proteins in an ambisense orientation. The S segment also contains the intergenic region (IGR) that separates the two open reading frames of N and NSs. The IGR forms a stem-loop structure and is important for the transcription termination of N- and NSs-coding RNA [13,14]. All three segments form an intramolecular panhandle structure, where the complementary 3′ and 5′-ends of the viral genomic RNA anneal together, similarly to other single-stranded negative-sense RNA viruses [15,16].

Because RVFV harbors a multipartite genome, packaging of all three genomic segments into a virion is required to form a fully infectious particle. The mechanism of RVFV genome packaging has been shown by several groups to be adaptable. For example, the creation of two- and four-segmented versions of RVFV is possible, suggesting the flexibility of packaging [17,18]. A codon-shuffled M segment can also be packaged, implying a weak requirement for an internal packaging signal [19]. On the other hand, the 5′-untranslated region (5′-UTR) of the M segment was shown to be important for packaging of the S and L segments [20]. Unlike other segmented RNA viruses, RVFV appears not to form supramolecular RNA complexes consisting of the three genomic segments during infection [19]. Moreover, genome packaging efficiency (i.e., the proportion of virus particles that are fully infectious) depends on cell type; arthropod cells, such as C6/36, show more efficient packaging than that of mammalian cells, like VeroE6 [21]. Furthermore, packaging genomic RNAs enhances the release of viral particles, suggesting the presence of a quality-control or surveillance mechanism [11]. Genome packaging in phleboviruses probably involves many host factors as well, and the complete mechanism remains unknown. Nonetheless, the interactions between the viral proteins Gn, RdRp, and N with vRNAs are critical within the process [9].

RVFV N is a 27 kDa RNA-binding protein that is essential in viral replication and packaging. N has a mainly α-helical structure and has been observed in crystallographic structures to adopt two major conformations, open and closed [22,23,24]. The crystallographic structure of the RNA-free dimeric form of N is in the closed conformation, where its RNA-binding cleft is occluded by the N-terminal helical arm [23]. Presumably, upon encounter with sequences or structures in target RNA, the N-terminal arm moves away from the RNA-binding cleft, shifting to the open conformation and allowing target RNA to interact. After the first binding event, the RNA-bound N cooperatively recruits other free N to oligomerize along the target RNA, which results in the RNA being largely or completely coated by the protein [25]. Formation of the N–RNA complex protects the vRNA and replication intermediates from nucleases and is essential for viral transcription, replication, and efficient packaging [26].

Because the RNA binding cleft is enriched in basic amino acids, a driving force for the interactions between N and RNA is thought to be electrostatic. It is worth noting that the crystallographic models of N–RNA complex do not reveal any base-specific contacts that would shed light on the sequence or structural preferences of the initial binding event [22,24]. However, it is possible that sequence-specific interactions between N and RNA bases have been overlooked because of the promiscuous RNA binding mode of N after the initial recognition event. We have previously reported that GAUU and pyrimidine/guanine motifs are RNA targets favored by N [27]. N–RNA interactions are required in many steps during viral replication, which makes them an attractive, potential therapeutic target for RVFV infection [28,29].

Previous studies revealed the importance of the interactions between viral glycoproteins and RdRp [11], N [30], and genomic and antigenomic RNAs [12] in the context of virion packaging. Likewise, the encapsidation of RNAs by N is expected to be a significant event, as enveloped RNAs are largely associated with N [23]. However, the landscape of N–RNA interactions in RVFV-infected cells remains unknown. We hypothesized that the N concentration qualitatively and quantitatively drives N–RNA interactions and this dictates which RNAs are packaged into nascent virions. In this study, we used crosslinking, immunoprecipitation, and sequencing (CLIP-seq) to characterize the interactions between N and RNAs in RVFV (MP-12)-infected cells. During the course of infection, N interacts with vRNAs as well as host protein-coding and noncoding RNAs, mostly in the cytosol. Somewhat unexpectedly, CLIP-seq, cell fractionation, and confocal analysis all revealed the presence of N in the nucleus of cells, where it interacts with various nuclear RNAs. In addition, we used multiple reaction monitoring mass spectrometry (MRM-MS) to precisely quantitate N expression in RVFV-infected cells. We found that the intracellular N expression increases exponentially early in infection and plateaus by 10 to 12 hpi. Additionally, N binds mostly to host RNAs early in infection, whereas the N–vRNA interactions predominate later in infection, which correlates with the increased specific infectivity of viral particles produced later during infection. Combining the precise measurements of RNAs and N with the high-throughput sequencing data, we show apparent requirements for production of infectious and incomplete viral particles and suggest a mechanistic advantage for collective infection, in line with recently proposed sociovirology-based spreading strategies [31].

## 2. Materials and Methods

### 2.1. Cells and Viruses

HEK293 and Vero cells were cultured in Dulbecco’s Modified Eagle Medium (DMEM; Gibco) supplemented with 10% fetal bovine serum (FBS; Gibco, 10437) and 1× penicillin-streptomycin (Gibco, 15140) and maintained at 37 °C and 5% CO_2_. RVFV (strain MP-12) working stocks were prepared by infecting Vero cells and harvesting supernatant. Small aliquots of the virus were stored frozen at −80 °C until use. HEK293 and Vero cells were obtained from American Type Culture Collection (ATCC). The parental stock of the virus was kindly provided by Dr. Brian Gowen (Utah State University, Logan, UT, USA). The viral titer was determined by flow cytometry and TCID50.

To infect cells, virus diluted in 2% FBS DMEM was placed on cells and incubated for 2 h at 37 °C and 5% CO_2_. Cells were washed, and the medium was replaced with fresh 2% FBS DMEM. Cells were incubated at 37 °C and 5% CO_2_ as indicated in each experiment.

### 2.2. Flow Cytometry

RVFV MP-12-infected cells were collected at 8 h post infection, washed twice with phosphate-buffered saline (PBS), and fixed with 4% formaldehyde in PBS. Fixed cells were incubated in Permeabilization Buffer (1% *w*/*v* bovine serum albumin (BSA), 0.1% *w*/*v* saponin, and 0.005% *w*/*v* sodium azide in PBS) for 10 min at room temperature and were spun down for 5 min at 500× *g*. The permeabilization step was repeated twice. Then, cells were incubated with anti-N antibody (BEI Resources, Manassas, VA, USA, NR-43188) diluted 1:2000 in Permeabilization Buffer for an hour at room temperature. After pelleting by centrifugation, cells were washed twice with Permeabilization Buffer. Alexa 488-conjugated secondary antibody (Invitrogen, Frederick, MD, USA, A11001) was diluted 1:2000 in Permeabilization Buffer, and cells were incubated with the antibody solution for 30 min at room temperature. Cells were washed twice with PBS for 10 min at room temperature.

Flow cytometry data were acquired by using Attune NxT Flow Cytometer (Invitrogen). FlowJo software (Becton, Dickinson & Company, Franklin Lakes, NJ, USA) was used for analysis, and the gating strategy was as follows: side scatter area and forward scatter area define cells, forward scatter width and forward scatter height define single cells. Infected cells are defined as single cells that exhibit greater fluorescent signal compared to mock-infected cells when forward scatter area is plotted against BL1 channel signal.

### 2.3. CLIP-Seq Lysate Preparation

Approximately 10^7^ MP-12-infected HEK293 cells (MOI = 0.1) were collected at various time points as follows; cells were washed twice with cold PBS, UV-crosslinked for 0.4 J/cm^2^ then 0.2 J/cm^2^, scraped, and pelleted. The pellets were flash frozen in liquid nitrogen and stored at −80 °C until use.

Cell pellets were lysed with 350 μL PXL Buffer (0.1% SDS, 0.5% sodium deoxycholate, 0.5% IGEPAL, and 1× mammalian protease inhibitor cocktail (Gold Biotechnology, Saint Louis, MO, USA) in PBS) by incubating on ice for 10 min. Then, 10 μL DNase RQ1 (Promega, Madison, WI, USA) and 3.6 μL diluted RNase T1 (Thermo Scientific, Waltham, MA, USA; to final concentration of 1:10,000) were added to each lysate. All tubes were incubated for 5 min at 37 °C, then digestion was quenched by placing tubes on ice. The lysates were spun for 20 min at 14,000 RPM 4 °C, and the clear supernatant was transferred to a clean tube. An amount of 20 μL of the supernatant was kept as size-matched control sample and was stored at −80 °C until use. Volumes of the remaining lysates were adjusted to 1 mL with PXL Buffer.

### 2.4. Immunoprecipitation

An amount of 50 μL of Protein A/G magnetic beads (Pierce, Rockford, IL, USA) was prepared with 20 μg of anti-N antibody (Maine Biotechnology Services, Portland, ME, USA, MAB240P, lot 1611296.1P) according to the manufacturer’s instructions. Lysate (1 mL) was added to the beads and incubated end-over-end at 4 °C overnight. The samples were washed twice each with LiCl Buffer (250 mM LiCl, 10 mM Tris-HCl pH 8.0, 1 mM EDTA, 0.5% sodium deoxycholate, and 0.5% IGEPAL), NaCl Buffer (50 mM Tris-HCl pH 7.4, 1 M NaCl, 1 mM EDTA, 0.1% SDS, 1% IGEPAL, and 0.5% sodium deoxycholate), and KCl Buffer (50 mM HEPES-KOH pH 7.5, 500 mM KCl, and 0.05% IGEPAL), then once with Dephosphorylation Buffer (1× Antarctic phosphatase buffer from NEB, Ipswich, MA, USA). Samples were incubated with 5 μL Antarctic phosphatase (NEB) in Dephosphorylation Buffer for 30 min at 37 °C, then washed twice each with Phosphatase Wash buffer (50 mM Tris-HCl pH 7.5, 20 mM EGTA, and 0.5% IGEPAL) and 1 mL PNK Buffer (−) DTT. End-labeling of RNA was carried out in PNK Buffer (50 mM Tris-HCl pH 7.5, 10 mM MgCl_2_, and 1 mM DTT) with 2 μL gamma-32P-ATP (Perkin Elmer, Boston, MA, USA) and 5 μL T4 polynucleotide kinase (Thermo Scientific). The reactions were incubated for 30 min at 37 °C. Finally, washes were performed once each with PNK Buffer (−) DTT, LiCl Buffer, NaCl Buffer, and KCl Buffer. The N–RNA complexes were eluted by adding 50 μL 1× NuPAGE LDS Loading Buffer (Invitrogen) to the beads.

### 2.5. Size Selection of Immunoprecipitated N–RNA Complexes

**Figure 1:**
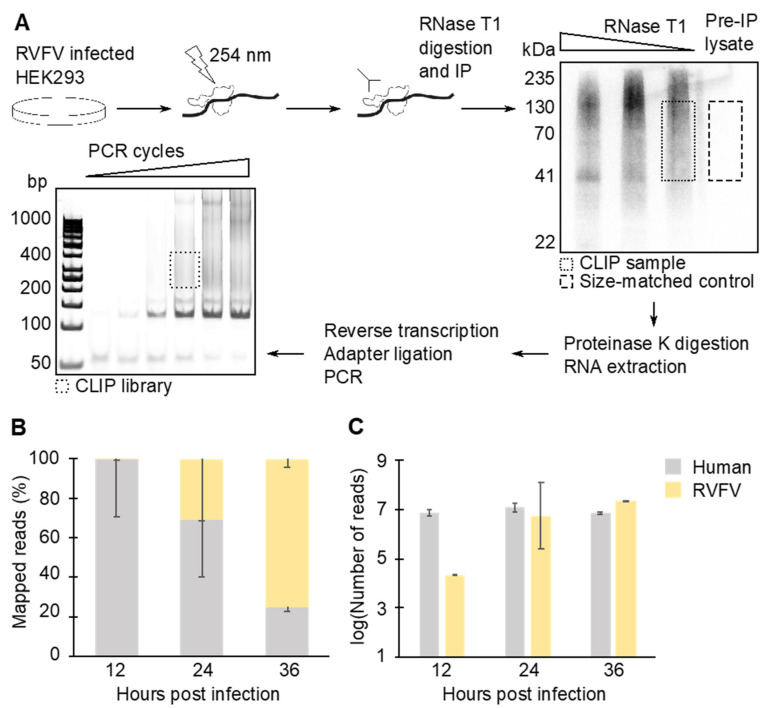
CLIP-seq library construction workflow and basic read statistics. (**A**) CLIP-seq libraries were prepared by crosslinking and immunoprecipitating N–RNA complexes. The size-matched samples were also prepared as a control by running pre-immunoprecipitation (IP) lysate on a gel and by purifying RNAs from the corresponding region. (**B**) CLIP-seq mapping statistics. N copurified mostly with human RNAs at 12 hpi, while at 36 hpi, more than 74% of copurified RNAs were viral. (**C**) Raw number of reads obtained from CLIP-seq libraries. Means and standard error of means (SEM) of two biological replicates are plotted in the graphs.

The IP samples were denatured 10 min at 70 °C and fractionated on 10% acrylamide Bis-Tris gel with NuPAGE MOPS-SDS buffer (Invitrogen). Protein–RNA complexes were transferred onto a nitrocellulose membrane with NuPAGE transfer buffer (Invitrogen) for 1 h, 30 V at room temperature. The region corresponding to N–RNA complex was excised. The RNA was eluted from the membrane by digesting the protein with proteinase K (Sigma, Saint Louis, MO, USA) in PK Buffer (4 mg/mL proteinase K, 200 mM Tris-HCl pH 7.5, 100 mM NaCl, 20 mM EDTA, and 2% SDS) for 30 min at 55 °C. RNAs were purified by phenol–chloroform extraction followed by ethanol precipitation. RNA concentration in each sample was determined by Qubit microRNA assay (Invitrogen). Size-matched samples, which are the stringent CLIP-seq controls recommended by the ENCODE project (https://www.encodeproject.org/ accessed 30 November 2021), were used in this study. These size-matched samples, which are not selected by IP of N, were prepared side-by-side with the IP samples (Figure 1A).

### 2.6. Sequencing Library Preparation and Deep Sequencing

CLIP-seq libraries were prepared by using NEBNext Ultra II Directional RNA library prep kit (NEB). PCR products were fractionated on a nondenaturing acrylamide gel, and the region corresponding to CLIP-library (200–400 base pairs) was excised. The fragments were eluted in Elution Buffer (500 mM ammonium acetate pH 8.0, 0.1% SDS, 1 mM EDTA, and 10 mM magnesium acetate) overnight at 37 °C. Finally, the DNA fragments were desalted by using DNA Clean and Concentrator kit (Zymo Research, Irvine, CA, USA). Sequencing reactions were carried out by Illumina HiSeq sequencer (Novogene, Tianjin, China).

### 2.7. Bioinformatics Analysis

All FASTQ files were first analyzed by FastQC (https://www.bioinformatics.babraham.ac.uk/projects/fastqc/ last accessed 30 November 2021, Babraham Bioinformatics, Cambridge, UK), and the adaptors were trimmed by using Cutadapt [32]. For the quality control, FastQC was run again to ensure adaptor removal. Then, reads were mapped to either human genome (hg38) or RVFV genome (GenBank: DQ375404.1, DQ380208.1, and DQ380154.1) by using RNA STAR [33] with default options for the paired-end sequencing, the strand flag (XS) output, and without soft-clipping. Mapped reads were indexed, position sorted, then deduplicated by using samtools markdup with -r and -s options (http://www.htslib.org/ last accessed 30 November 2021). For peak calling analysis of host RNAs, PEAKachu (https://github.com/tbischler/PEAKachu accessed 30 November 2021) was used with two biological replicate sequencing data and the size-matched control data, and with the default parameters, except for --paird_end and --max_insert_size 150 options. Peaks were then annotated by using UROPA [34] with the default parameters, except for “strand”:“same” “internals”:“1.0” and “show_attributes”;“all” options. For the analysis of reads that aligned to MP-12 genome, mapped and deduplicated reads were visualized by using Integrated Genome Viewer [35] (version 2.8.10). FastQC, Cutadapt, RNA STAR, and PEAKachu were run through the Galaxy server [36].

### 2.8. Immunofluorescence and Confocal Microscopy

Coverslips were prepared in 6-well plates by incubating with poly-D-lysine (Gibco) for an hour at room temperature. The coated coverslips were then washed three times with sterile water and dried. HEK293 cells were grown on the coverslip and infected with RVFV MP-12 as described above. On the day of harvest, cells were fixed with 4% (*v*/*v*) formaldehyde in PBS, then permeabilized with 0.1% (*v*/*v*) Triton-x100 in PBS. Cells were blocked by incubating with Blocking Buffer (2% (*w*/*v*) BSA in PBS) and were stained with anti-N antibody (BEI Resources, NR-43188) diluted 1:2000 in Blocking Buffer overnight at 4 °C. Cells were washed three times with Blocking Buffer, and incubated with Alexa 488-conjugated secondary antibody (Invitrogen, A11001) diluted 1:8000 in Blocking Buffer for 2 h at room temperature. Slides were washed three times with PBS, mounted with ProLong Diamond with DAPI (Invitrogen), and cured for 24 h at room temperature. Individual z-slice images were obtained by using Zeiss Laser-Scanning Microscope 880 and analyzed by using Zen Lite (Zeiss, White Plains, NY, USA). All confocal microscopy images presented are single focal planes of z-stacks and not maximum-intensity projections (MIP). Even though MIP is commonly used and accepted, the evaluation of single-focal-plane images was most suitable for this study. MIP combines multiple z-slices to create a two-dimensional image, which potentially leads to compromised resolution in x- and y-directions [37]. By assessing one focal plane only, false-positive signals that could arise from the integration of lower and upper planes can be eliminated. This enables simple assessment of the xy-spatial relationships between the nuclear signal (DAPI) and the anti-N antibody signal (Alexa 488).

### 2.9. Western Blot

HEK293 cells were transfected with a FLAG-N expression plasmid by using Lipofectamine 2000 (Invitrogen). At 24 hpi, cells were harvested by trypsinization and centrifugation. After two washes with PBS, cells were swollen with Hypotonic Lysis Buffer (20 mM HEPES pH 7.4, 10 mM NaCl, 3 mM MgCl_2_, and 1× Protease inhibitor (Gold Biotechnology)) and lysed with 27G needle in presence of 25 μg/mL digitonin. Digitonin is a mild detergent that has been shown not to lyse nuclei [38]. Insoluble, nuclear fraction was pelleted by centrifugation for 10 min at 1500× *g*, washed with PBS, and lysed with NuPAGE LDS Sample Buffer (Invitrogen). Soluble, cytoplasmic fraction was spun down for 15 min at 10,000× *g*, and the clear supernatant was kept for analysis. Samples were electrophoresed under reducing and denaturing conditions, and transferred onto a PVDF membrane overnight. Antibodies used for the blotting were anti-FLAG (Sigma, F3165, 1:2000), anti-GAPDH (BioLegend, 607901, 1:1000), anti-histone H2B (BioLegend, 606301, 1:1000), anti-mouse (Sigma, A2554, 1:6000), and anti-rat (BioLegend, 405405,1:2000).

### 2.10. Strand-Specific RT-qPCR

The strand-specific RT-qPCR for RVFV was designed to use TaqMan chemistry [39,40]. The regions targeted by each primer were chosen based on the transcription end-sites described previously [13,14]. The primer sequences are summarized in Supplemental Tables S1 and S2. Single-stranded DNAs were synthesized by Integrated DNA Technologies (IDT; Coralville, AI, USA), and the TaqMan probe was synthesized by Sigma. First, standard RNAs were in vitro transcribed from double-stranded PCR fragments possessing T7 promoter and viral sense, anti-sense, and transcript sequence. Transcription reactions were carried out by using TranscriptAid T7 High Yield Transcription Kit (Thermo Scientific), and products were gel-purified. The concentration of each standard was calculated based on 260 nm absorbance measured by Nanodrop 2000 (Thermo Scientific) and the theoretical extinction coefficient calculated by OligoAnalyzer Tool (IDT). To generate RT-qPCR standard curves, 0.5 μL of diluted RNA template was mixed with 0.5 mM dNTPs, 100 nM reverse transcription primer, 5 mM DTT, 1× SuperScript IV Buffer, and 50 units of SuperScript IV (Invitrogen). Reverse transcription (RT) reaction was carried out for 10 min at 50 °C, then was terminated by heating samples 10 min at 80 °C. qPCR reactions (20 μL) were assembled with TaqMan Gene Expression Master Mix (Applied Biosystems, Waltham, MA, USA), 0.5 μM each forward and reverse primer, 50 nM RVFV probe, and 2 μL RT reaction. The qPCR reactions were carried out by StepOne Real-Time PCR System (Applied Biosystems), and cycle conditions were as follows: 2 min at 50 °C, 10 min at 95 °C, then 40 cycles of 15 s at 95 °C and 1 min at 60 °C. All qPCR reactions were performed in duplicate. StepOne Software (v2.3, Applied Biosystems) was used for data analysis. Copy numbers of viral protein-coding RNAs were calculated by subtracting number of antigenomic segment RNAs (and the genomic S segment for NSs) from total number of antisense RNAs (and the S sense RNA for NSs).

### 2.11. Purification of RNAs from Cell Culture and Cell Culture Supernatant

Cellular RNAs were purified by using TRIzol reagent (Invitrogen) and RNA Clean and Concentrator Kit (Zymo Research). An amount of 50 ng of purified total RNA was used in 5 μL reverse transcription reaction as described above. Viral RNAs released in cell culture media were purified as follows. First, cell culture medium was spun down for 10 min at 1000× *g*, and clear supernatant was transferred to a clean tube. An equal volume of acidic phenol–chloroform (Invitrogen) was added, and the tube was vortexed then spun down for 20 min at 20,000× *g*. RNAs in the aqueous phase were purified by using RNA Clean and Concentrator Kit (Zymo Research).

### 2.12. Preparation of 15N-Labeled N

Recombinant N protein was expressed in BL21 (DE3) pLysS cells (Promega) by first, preculturing cells in the LB broth containing 50 μg/mL ampicillin overnight at 37 °C. The preculture was pelleted and was resuspended in 6 mL M9 medium (per one liter: 60 g divalent sodium phosphate, 30 g monovalent potassium phosphate, and 5 g NaCl) supplemented with Trace Elements (50 μg/mL EDTA, 8.3 μg/mL ferric chloride hexahydrate, 0.84 μg/mL zinc (II) chloride, 0.13 μg/mL copper (II) chloride dihydrate, 0.10 μg/mL cobalt (II) chloride hexahydrate, 0.10 μg/mL boric acid, and 0.016 μg/mL manganese (II) chloride hexahydrate), 1 mM MgSO_4_, 0.3 mM CaCl_2_, 1 μg/mL thiamin, 1 μg/mL biotin, and 1 mg/mL ^15^NH_4_Cl. Then, 1 mL cells was added to 500 mL M9 medium containing 50 μg/mL ampicillin and incubated at 37 °C till OD600 reached 0.5. Next, IPTG was added to 5 mM, and the culture was incubated for 16 h at 23 °C. Cells were pelleted by centrifugation at 5000 RPM, 4 °C for 20 min in F9-6x1000 LEX rotor (Thermo Scientific) and resuspended in 50 mL E.coli Lysis Buffer (50 mM Tris-HCl pH8, 300 mM NaCl, 5% Glycerol, 5 mM imidazole, 23 ng/mL PMSF, 21 ng/mL TLCK, 21 ng/mL TPCK, 3.3 ng/mL leupeptin, and 3.3 ng/mL lima bean). Twenty milligrams lysozyme was added to cells, and after 20 min incubation at 4 °C, cells were lysed with a cell disruptor. The lysate was centrifuged for 45 min at 36,000 RPM, 4 °C in Ti45 rotor (Beckman, Indianapolis, IN, USA), and filtered through a 0.45 μm membrane.

The following purification steps were conducted at 4 °C. ^15^N-labeled N was bound to Profinity Ni-IMAC resin (BioRad, Hercules, CA, USA) by running the lysate through a gravity-flow column. The resin was washed with Wash Buffer (50 mM Tris-HCl pH 8, 500 mM NaCl, 10% glycerol, and 10 mM imidazole), and the protein was eluted with 10 mL Elution Buffer (50 mM Tris-HCl pH 8, 500 mM NaCl, 10% glycerol, 300 mM imidazole, and the protease inhibitors as listed in E.coli Lysis Buffer). The eluate was concentrated down by centrifugation in Amicon 10 MWCO column (Sigma). Aliquots of purified proteins were frozen with liquid nitrogen and were stored at −80 °C.

### 2.13. Sample Preparation for MRM-MS

HEK293 cells were infected with RVFV MP-12 (MOI = 1 or 2) as described above. Cells were washed twice with PBS, scraped off the plate, transferred to a tube, and pelleted by centrifugation for 10 min at 1000× *g*. Cell pellets were resuspended in PBS and lysed with Urea Lysis Buffer (50 mM Tris-HCl pH 8.3, 75 mM NaCl, and 8 M urea). Lysates were briefly sonicated to reduce viscosity and spun down for 20 min at 10,000× g. The total protein content was estimated by using Bradford assay. An amount of 0.1 μg ^15^N-labeled N was added per 30 μg total protein. Samples were reduced with DTT, denatured in NuPAGE LDS Loading Buffer (Invitrogen) by heating 10 min at 70 °C, and fractionated on SDS-containing acrylamide gel. To avoid distortion, samples of less than 50 μg were loaded per lane of a minigel. Fractionated proteins were stained with Coomassie, and a band corresponding to N was excised, minced, and transferred to a clean tube. The gel pieces were destained twice for 20 min at 37 °C, by incubating in Destaining Buffer (50 mM ammonium bicarbonate and 50% (*v*/*v*) acetonitrile). Next, the gel pieces were incubated for 10 min at 55 °C in Reducing Buffer (50 mM ammonium bicarbonate and 50 mM DTT) and for 1 h at room temperature in Alkylation Buffer (50 mM ammonium bicarbonate and 14 mM iodoacetamide). The gel pieces were washed twice in Destaining Buffer for 15 min at 37 °C and dehydrated in acetonitrile. Then, acetonitrile was removed, and all samples were air dried. MS-grade trypsin (Pierce) containing Digestion Buffer (50 mM ammonium bicarbonate and 1 mM CaCl_2_) was added to the gel pieces to rehydrate. Extra Digestion Buffer was added to cover gel pieces, and samples were incubated overnight at 37 °C. Peptide-containing supernatant was transferred to a clean tube, and desalted by using Peptide Desalting Spin Column (Pierce). The eluate was dried in a SpeedVac, and the pellet was stored at −80 °C until analysis.

### 2.14. MRM-MS for RVFV N

Initial screening for peptides ideal for MRM-MS was performed using an Agilent 6520 quadrupole time-of-flight mass spectrometer coupled to Agilent 1260 UPLC (Agilent, Santa Clara, CA, USA). Native or ^15^N-labeled recombinant N protein was digested with trypsin, and peptides were separated using AdvanceBio Peptide Mapping column (2.7 μm, 120 Å, 2.1 × 100 mm; Agilent) over 90 min with a gradient of 3–50% Solvent B (acetonitrile with 0.1% formic acid) and Solvent A (water with 0.1% formic acid). Peptides were identified using Morpheus software [41] with a precursor mass tolerance of 1.2 Da, product mass tolerance of 0.5 Da, and maximum FDR of 1%. For MRM, an Agilent 6460 Triple Quadrupole mass spectrometer in-lined with Agilent 1200 binary LC system was used for analysis. The peptide mapping column was used for the separation of peptides. The LC gradient used is as follows: increase Solvent B from 5% to 30% over 2 min, increase Solvent B from 30% to 40% over 5 min, increase Solvent B from 40% to 85% over one minute, hold at 85% Solvent B for 3 min, and re-equilibrate back to 5% Solvent B over 3 min. The flow rate was set at 0.3 mL/minute, and the column was kept at 50 °C. The injection volume was 5.00 μL. To avoid carryovers, blank injections were performed between different samples. On the mass spectrometer, the gas temperature was set at 325 °C, sheath gas flow was set at 11 L/minute, and capillary voltage was set at 4000 V. For MRM, the y9 ion of the peptide (AILDAHSLYLLQFSR) was monitored. The transition of endogenous N monitored was Q1 = 874.1 m/z to Q3 = 1126.4 m/z, with fragmenter = 180 V and collision energy = 45 V. The transition of ^15^N-labeled N monitored was Q1 = 884.6 m/z to Q3 = 1139.6 m/z, with fragmenter = 180 V and collision energy = 43 V. Dwell for all transitions was set at 100. MRM area under the curve (AUC) was calculated by using Mass Hunter software (Agilent). To estimate the absolute amount of endogenous N in the sample, the ratio of the amount spiked-in to AUC for ^15^N-labeled N was calculated. Then, AUC obtained from endogenous N was multiplied by the ratio calculated from ^15^N-labeled N.

## 3. Results

### 3.1. N Binds Viral and Cellular RNA during Infection

N participates in various steps in the viral life cycle. N may regulate transcription and replication by acting as a chaperone for vRNAs. In addition, interactions with N are required for RNAs to be packaged in nascent virions. We sought to characterize the N–RNA interactions in RVFV MP-12-infected cells over the course of infection using CLIP-seq. RVFV-infected HEK293 cells were UV-irradiated, and the N-RNA complexes were immunoprecipitated (Figure 1A). During the library preparation, RNase T1 concentration was optimized to produce RNA fragments with sizes ideal for the subsequent sequencing reactions. The authentic N-RNA complex purified from an intact virion is known to be nuclease resistant [23]. Thus, the RNase T1 digestion was performed in presence of several detergents. The overdigested N-RNA complex showed a distinct band around 41 kDa (Figure 1A), which is in good agreement with the expected supershift of the 27 kDa protein. The 41 kDa band corresponds to an N monomer crosslinked to a small piece of RNA, which provides a reference for the size exclusion step [42]. To account for cellular ribonucleoprotein (RNP) complexes that nonspecifically bind to beads during immunoprecipitation (IP), the pre-IP, size-matched control [43] was prepared side-by-side with the experimental sequencing libraries. The sequences obtained from the control library were subtracted from the experimental sequencing results at the time of bioinformatics analysis. The RNAs purified from the RNP complexes were subjected to deep sequencing followed by bioinformatics analysis. A total of 125 million mapped reads were obtained from biological duplicate sequencing libraries and the size-matched control library combined. At 12 hpi, the majority of RNA copurified with N was cellular RNA (Figure 1B). The proportion of reads that aligned to vRNA species increased as the infection time progressed. At 36 hpi, the vRNA reads exceeded 74% of total mapped reads. Notably, the raw number of reads that were identified as host RNA stayed consistent at all time points tested (Figure 1C). Therefore, the increase in the ratio of viral:human RNA copurified with N at later time points was due to increased N–vRNA interactions.

### 3.2. N Binds Host Cell Protein-Coding Transcripts

**Figure 2:**
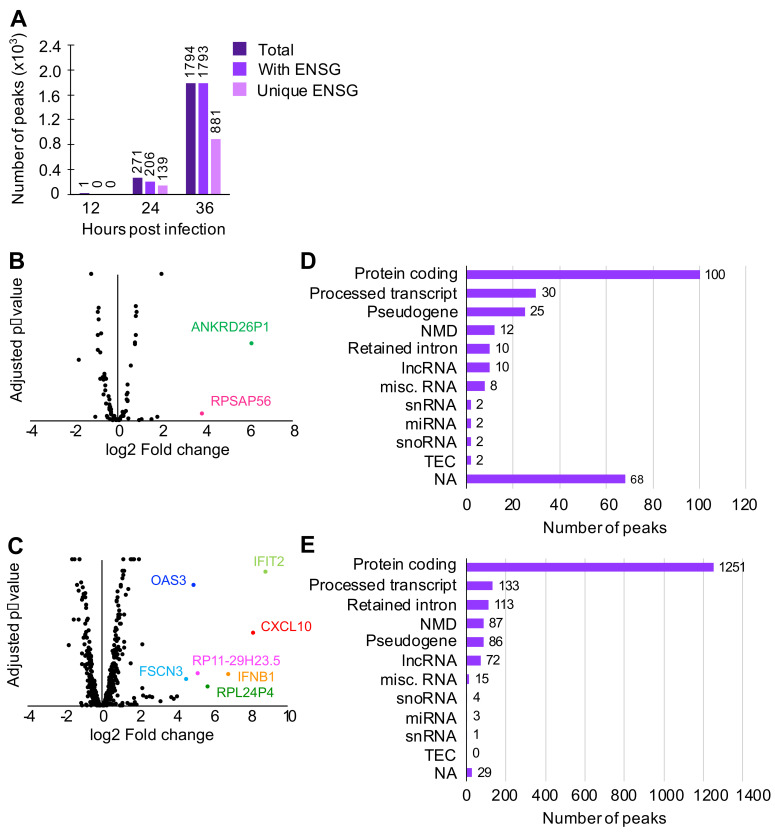
Peak calling analysis of reads aligned to human RNAs. To find regions on transcripts to which N was highly bound, peak calling analysis was conducted. (**A**) Number of peaks with Ensembl gene ID (ENSG). (**B**,**C**) Crossover analysis between CLIP-seq (this study) and transcriptomics of HEK293 cells infected with MP-12 [3]. CLIP-seq peaks were identified on transcripts that are neither up- or downregulated, with the following exceptions. *ANKRE26P1* and *RPSAP56* at 24 hpi (**B**) and *IFIT2*, *CXCL10*, *OAS3*, *IFNB1*, *RP11-29H23.5*, *RPL24P4*, and *FSCN3* at 36 hpi (**C**). (**D**,**E**) Transcript biotypes of each peak found at 24 hpi (**D**) and 36 hpi (**E**). NMD = nonsense mediated decay. lncRNA = long noncoding RNA. miscRNA = miscellaneous RNA that includes ribosomal RNA and vault RNA. snRNA = small nuclear RNA. miRNA = microRNA. snoRNA = small nucleolar RNA. TEC = to be experimentally confirmed. ESTs (expressed sequencing tags). NA = not applicable (no transcript biotype assigned to the peak).

Various host RNAs, including those that are expressed in response to viral infection, are expected to be present in the cytoplasm. To characterize host RNAs that copurified with N, peak calling analysis was conducted using PEAKachu (https://github.com/tbischler/PEAKachu accessed 30 November 2021), followed by peak annotation by UROPA [34]. Early in infection, N was observed to bind primarily to host RNAs without strong clustering at specific loci, although one peak corresponding to an uncharacterized region of the human genome was found at 12 hpi (Figure 2A). At later time points, however, an increasing number of peaks were identified, namely, 271 peaks at 24 hpi and 1794 peaks at 36 hpi. At these time points, approximately 50% of total peaks were associated with unique Ensembl gene IDs (ENSGs). PANTHER gene ontology analysis (http://geneontology.org/ accessed 30 November 2021) of these ENSGs revealed that transcripts for proteins that participate in metabolic processes and protein biosynthesis (ribosomal proteins and translation initiation/elongation factors) were enriched at 24 hpi. At 36 hpi, in addition to the processes found at 24 hpi, chromosome organization (histone proteins) and protein folding (chaperones and chaperonins) were enriched. Although antiviral protein transcription is upregulated and therefore transcripts are more abundant during infection, none of the time points indicated with statistical significance that N specifically targeted transcripts coding for antiviral proteins as a group.

Published crystal structures of N show a positively charged cavity that interacts with the negatively charged RNA backbone, suggesting that N, in this conformation, does not recognize particular RNA sequences [22,23,24]. However, if N–RNA interactions were entirely nonspecific, it would be expected that N would bind to RNAs that are most abundant during infection. To test this, we compared CLIP-seq peaks to our transcriptome data of RVFV MP-12-infected cells [44] (Figure 2B,C). At both 24 and 36 hpi, the majority of peaks found in CLIP-seq did not correlate with significantly upregulated or downregulated transcripts, although several peaks were found on transcripts that are upregulated during viral infection, such as the pseudogenes *ANKRD26P1* and *RPSAP56* at 24 hpi, and cellular viral response genes *IFIT2*, *IFNB1*, *CXCL10*, and *OAS3* at 36 hpi.

To further characterize the host transcripts bound by N, 24 and 36 hpi peaks were sorted into transcript biotypes (Figure 2D,E). At both time points, protein-coding transcripts, which are expected to be less highly structured and therefore more available for protein interaction, produced the largest number of peaks. A few peaks belong to RNAs that are known to contain both highly ordered structures and single-stranded regions, such as ribosomal RNAs and transfer RNAs.

**Figure 3:**
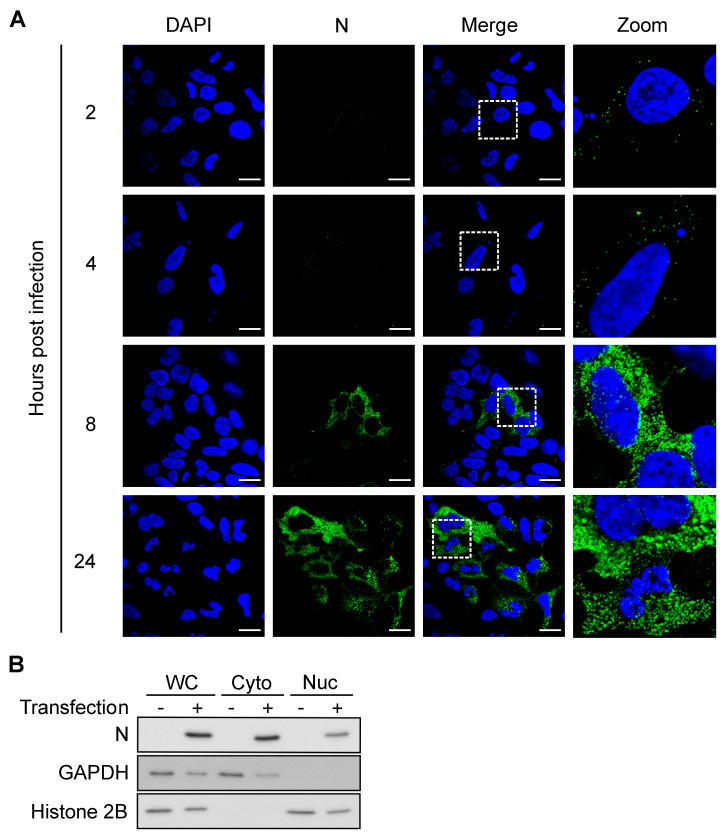
N localizes to cytoplasm and nucleus after 8 hpi. (**A**) z-slice images from confocal microcopy analysis. MP-12-infected HEK293 cells were fixed at indicated time points and stained with anti-N antibody. Scale bars = 10 μm. (**B**) Western blot. HEK293 cells were transfected with a recombinant FLAG-N expression plasmid and fractionated into the cytoplasmic (Cyto) and the nuclear (Nuc) fractions. WC = whole cell. GAPDH = cytoplasmic marker. Histone 2B = nuclear marker.

Notably, some processed transcripts, including transcripts containing a retained intron(s), small nuclear RNAs (snRNAs), and small nucleolar RNAs (snoRNAs), also produced peaks, which was unexpected since these RNAs are known to mainly localize to the nucleus. Since RVFV replicates in the cytoplasm, interactions between N and nuclear RNAs would require either translocation of N to the nucleus or of nuclear RNAs to the cytoplasm. We investigated N localization by confocal microscopy and found that a fraction of N was indeed present in the nucleus as early as 8 hpi (Figure 3A). During infection, RVFV expresses NSs, a nuclear protein that is known to change localization of a host poly(A)-binding protein [45]. We asked if the presence of N in the nucleus was a result of altered nuclear permeability caused by NSs. To test this, HEK293 cells were transfected with an N expression vector and were fractionated into nuclear and cytoplasmic fractions. N was detected in both cytoplasmic and nuclear fractions in the absence of NSs, indicating that N itself can translocate into the nucleus (Figure 3B). Taken together, these data demonstrated that N is able to bind to host RNAs both in the cytoplasm and in the nucleus.

### 3.3. N Exhibits High- and Low-Density Binding Regions on Viral Sense and Antisense RNAs

**Figure 4:**
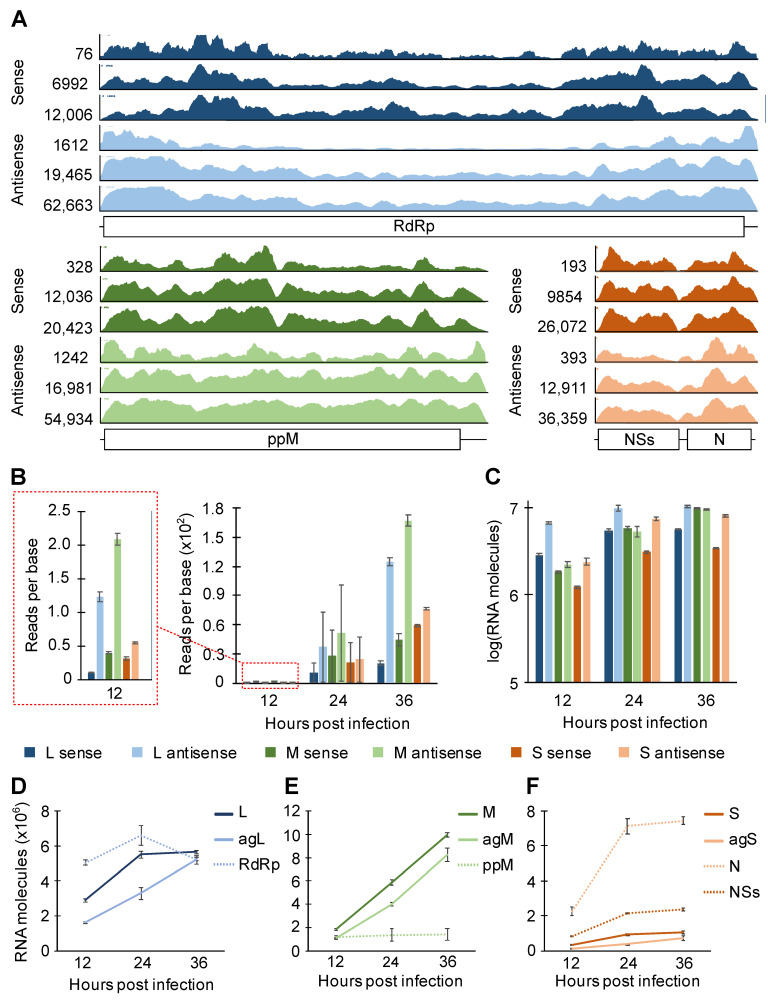
CLIP-seq read coverage on RVFV MP-12 sense/antisense segment and quantitation of intracellular viral RNAs. (**A**) Read coverages on RVFV RNAs are visualized. From top to bottom, 12, 24, and 36 hpi. The scales of the y-axes are defined by the height of the tallest peak at each time point. ppM = polyprotein M. (**B**) Statistics of reads-per-base CLIP-seq coverage. (**C**–**F**) Stranded RT-qPCR quantification of intracellular RNAs harvested from HEK293 cells infected with RVFV (MP-12) at MOI of 0.1. Means and SEMs of n = 2 biological replicates are plotted. L = L genome. agL = L antigenome. M = M genome. agM = M antigenome. S = S genome. agS = S antigenome. RdRp, ppM, N, and NSs signify coding transcripts for these proteins.

To characterize vRNAs that were copurified with N, the read coverage on each sense/antisense vRNA was visualized by Integrative Genomics Viewer [35] (Figure 4A). A few common features were shared among the three RVFV segments. For example, there were more pronounced peaks (regions with high read density) and valleys (regions with low read density) in sense versus antisense RNA. By visual inspection, we also found the valley regions to have A-rich sequences and the peak regions to have GU-rich sequences. Although the number of reads that mapped to a given segment varied greatly, the overall peak/valley profile was similar across all three time points tested. Thus, N exhibits preferential binding within a sense and antisense segment independent of progress in infection.

The analysis of read coverage across the three different segments showed, surprisingly, that a greater number of reads were mapped to the antisense segments (note that the y-axes on Figure 4A are scaled to the tallest peak represented). It is important to note that the relatively short CLIP-seq reads cannot readily distinguish viral protein-coding mRNAs and antigenomic RNAs (and the S genomic segment and the NSs transcript) unless a read was mapped to the 3′-UTR, which would be present only in antigenomic RNAs (and the S genomic RNA in case of S versus NSs). Therefore, we defined the L and M *sense* RNAs as the genomic L and M segments, respectively. For the S segment, the sense RNAs are the combined read counts for the genomic S segment and the NSs protein-coding transcript. The *antisense* RNAs are defined as the combination of the antigenomic segments and the protein-coding transcripts (RdRp for L, the polyprotein for M, and N for S). By normalizing the read counts to the number of nucleotides in each segment, we found that N copurified with more antisense RNAs than their counterpart sense RNAs in all segments (Figure 4B). If antisense RNAs were expressed more robustly than sense RNAs were, N would be expected to bind to these high-concentration RNA species at higher frequency. To investigate the expression of sense and antisense vRNAs, each vRNA species was quantitated using strand-specific RT-qPCR (Supplemental Figures S1–S3 and Supplemental Table S2). At all time points tested, approximately twice as many L and S antisense RNAs were present than their sense RNA counterparts (Figure 4C). The M segment was the exception, where roughly equal amounts of sense and antisense RNAs were observed. Thus, since N association with M segment RNAs does not mirror their relative abundances, these data suggest N exhibits higher affinity toward M antisense RNA than toward M sense RNA.

We also estimated viral mRNA expression by using RT and qPCR primers that target different regions of vRNAs to specifically differentiate viral mRNAs from other vRNA species (Supplemental Figure S1). For the L and S segments, antisense protein-coding transcripts (RdRp and N) were robustly expressed throughout infection (Figure 4D,F), contributing to the high counts for antisense molecules represented in Figure 4C. The expression of N protein-coding transcript is especially striking as 10- to 19-fold more N transcript was expressed than the antigenomic S RNA (Figure 4F). Repeated analyses of each vRNA transcript showed that the expression of viral genomic and antigenomic RNAs was more consistent between different passage numbers of cells than the expression of viral protein-coding transcripts was (Supplemental Figure S4). In summary, the pattern of N binding to sense and antisense RNAs was similar through different time points of infection, and N showed a general preference for the antisense M segment over the sense segment.

### 3.4. N Concentration Increases Exponentially Early during Infection

**Figure 5:**
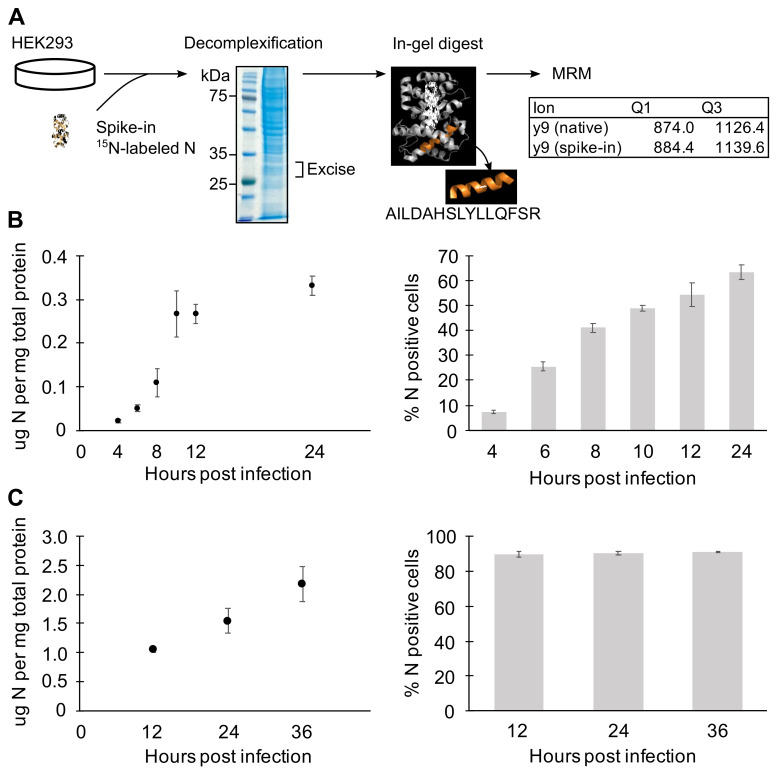
Quantitation of N protein expression by MRM-MS. (**A**) MRM-MS sample preparation workflow. (**B**,**C**) Quantitation of N protein expression by MRM-MS (left panels), and the flow cytometry analysis of N-expressing cells in corresponding samples (right panels). HEK293 cells were infected with MP-12 at MOI = 1 to characterize N levels arising from single infections (**B**) and later time points with MOI = 2 to observe effect of multiple/collective infection (**C**). Means and SEMs of n = 2 or 3 biological replicates are plotted.

Previous studies have shown that RVFV (Clone 13) infection can produce incomplete particles that include only one or two viral genomic segments [19]. To assess whether a low concentration of N at the early stages of infection might contribute to incomplete packaging, we used multiple reaction monitoring mass spectrometry (MRM-MS) to quantify N at several time points post infection. MRM-MS is a targeted proteomics method that enables accurate quantitation of N protein levels in cells without relying on antibody detection. RVFV-infected HEK293 cells were harvested at various time points, and total protein concentration was determined using the Bradford assay. As an internal control, 0.1 μg ^15^N-labeled N was added per 30 μg total protein, and samples were prepared for LC-MS/MS analysis (Figure 5A).

At earlier time points when cells received an average of one viral particle (MOI = 1), N expression increased 2-fold every two hours up until the plateau observed around 0.3 μg N per mg of total protein (Figure 5B). In our system, it typically takes 10 to 12 h for all cells that were initially infected with virus to reach the threshold level of N expression that can be detected by flow cytometry. Here, approximately 55% of the cells were N-positive by 12 h (Figure 5B). Among the cells that received virus(es), N expression followed a biphasic pattern of initial exponential increase followed by a linear phase. At early time points and low MOI, the observed exponential increase of N expression is due to increasing intracellular concentration since the major release of nascent particles and spreading infection falls outside the 10 hpi time window [19].

To characterize N expression under conditions in which cells are collectively infected, the N protein level was quantitated with cells infected at higher MOI and later time points (Figure 5C). Between 12 and 36 h, all cells are expected to be infected with multiple virus particles from the inoculum and newly released viruses. Accordingly, the flow cytometry analysis of the cell populations showed about 90% of cells expressing N (Figure 5C). Even with extended time over 24 h and with higher MOI, the N expression increased exponentially only up to 12 hpi. Because N mRNA concentrations were shown to remain high at later time points (Figure 4F), this could reflect either that cellular translation was impaired after 10 hpi as a response to viral infection [17,46] or that higher N concentrations promoted autoregulatory translational suppression [47]. In summary, N protein expression exponentially increases early in the infection and is kept at steady state by unknown mechanisms.

### 3.5. Cells Produce More Infectious Virus Later during Infection

**Figure 6:**
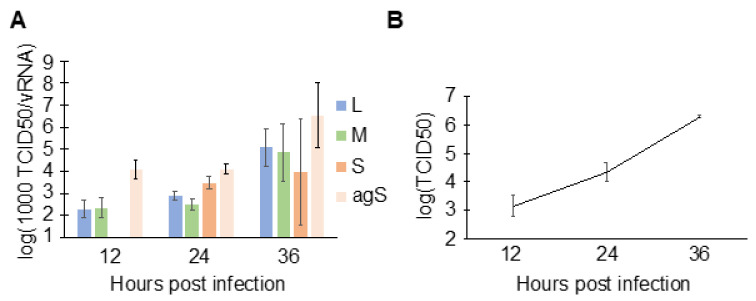
Specific infectivity of viral particles. (**A**) HEK293 cells were infected with MP-12 (MOI = 0.1), and the virus-containing cell culture media were harvested at indicated time points. Viral genomic RNA and the antigenomic S segment in media were quantified using strand-specific RT-qPCR, and TCID50s were measured to determine net infectious units. To express values in log scale, the ratio of TCID50/vRNA was transformed by multiplying by 10^3^ (1000 TCID50/vRNA). Means and SEMs of n = 2 biological replicates are plotted. The star indicates that RNA in the sample was below quantification range. (**B**) Viral growth curve at time points shown in (**A**). HEK293 cells were infected with MP-12 at MOI = 0.1. Means and SEMs of n = 2 biological replicates are shown.

To understand the consequence of N binding to host RNAs at early time points (Figure 1B), the specific infectivity of viral particles with respect to each genomic segment was characterized. First, HEK293 cells were infected with RVFV, and the supernatant containing viral particles was harvested at indicated time points. The supernatant was used to extract viral RNA for RT-qPCR, as well as to determine the end-point titration (median tissue culture infectious dose; TCID50). By taking the ratio of the end-point titration to vRNA, the number of fully infectious particles per vRNA molecules (TCID50/vRNA) can be estimated. Growth of RVFV MP-12 on HEK293 cells has previously been characterized [48]. In addition to the three viral genomic segments, the number of antigenomic S (agS) segments was also quantitated since a previous report showed that this RNA is also packaged [12]. At 12 hpi, principally the L, M, and agS were packaged (Figure 6). The S segment concentration fell below the quantification limit at 12 hpi, which is consistent with the low intracellular concentration estimated at the same time point (Figure 4F). Then, at 36 hpi, the specific infectivity increased by 50- (M segment) to 260-fold (L segment) compared to those at 12 hpi. The increased specific infectivity at 36 hpi suggests that a greater proportion of fully infectious and fewer incomplete particles were present in the cell culture media, demonstrating increased packaging efficiency. RVFV-infected cells are known to release vRNAs in exosomes [49,50], and such vRNAs may contribute to the overestimation of vRNAs in the RT-qPCR assay. Thus, we immunoprecipitated viral particles in the media of infected cells using anti-Gn and anti-Gc antibodies and compared the quantities of vRNAs that are enveloped (with membrane expressing Gn/Gc) and unenveloped (free RNP complexes and exosomes). At 14 hpi, approximately 90% of the L genomic segment in the supernatant was packaged within viral particles (unpublished work), indicating that the amount of vRNAs released in exosomes and as free RNPs was relatively minor. Taken together, more infectious viral particles were produced later in infection, which correlates with the N–vRNA interactions observed in RVFV-infected cells (Figure 1B).

## 4. Discussion

In this study, we used quantitative assessment of N protein and viral RNA levels along with a complete cataloging of N–RNA interactions to address knowledge gaps in the molecular virology of RVFV infection. In particular, these results elucidate the extensive interaction network of N with host and viral RNAs, and the dynamics of N and vRNA levels as a predictor of nascent viral infectivity, and suggest that the formation of incomplete virus particles and collective infection of cells are inherent in the overall virus spread strategy of RVFV.

### 4.1. Consequences of N Binding to Host RNAs

We conducted CLIP-seq to characterize the interactions between N and RNAs during RVFV infection. During the first replication cycle (within 12 h of infection with MOI of less than 1), N binds mostly to host RNAs even though robust expression of vRNAs is observed (Figure 1B,C). Among host RNAs, N associates largely with protein-coding transcripts, consistent with the notion that N localizes mainly to the cytoplasm and binds weakly structured RNAs. Our investigation revealed, however, a fraction of N was found in the nucleus and bound nuclear RNAs. Nuclear localization of N has been observed for some other RNA viruses that replicate in the cytoplasm [51,52], but this has not been observed in the phleboviruses. Because no nuclear localization signal has been characterized in RVFV N, and the ratio of nuclear/cytoplasmic N was rather low (Figure 3A), N likely enters the nucleus by diffusion through nuclear pores [53].

We previously reported that RVFV infection changes the host pre-mRNA splicing landscape [44]. Splicing requires direct interactions between the spliceosome and pre-mRNAs and occurs co-transcriptionally within the nucleus [54]. Therefore, since N binds abundantly to host RNAs and is localized partially to the nucleus, it is plausible that N could interfere with the function of splicing machinery by direct competition. We found instances of N binding to intron-containing RNAs in the CLIP-seq analysis, although we did not observe broad overlap between bound host RNAs and those that were determined by RNA-seq analysis to be differentially spliced during infection. These results suggest that N does not cause generalized splicing changes through direct, competitive interactions with host pre-mRNA transcripts, although other interactions with host splicing machinery cannot be ruled out.

N was found to interact with several transcripts that are upregulated during RVFV infection, including *IFNB1*, *IFIT2*, and *OAS3* (Figure 2B,C). *IFNB1* is a well-studied cytokine that is released in response to a viral infection [55]. *IFIT2* is expressed in response to the interferon-alpha/beta signaling and is characterized as an antiviral protein in influenza A infection and West Nile virus [56,57]. *OAS3* expression is also induced through the interferon signaling pathway, and the protein acts against viral infection [58,59]. The interactions between these transcripts and N may be due to the increased concentration of the host transcripts, or N may target them by recognition of RNA sequence or structural motifs that have not been characterized. It remains unknown whether the interactions between N and these antiviral protein-coding transcripts affect the protein levels, although this would constitute an intriguing strategy to evade host antiviral responses. It is notable that nucleocapsid proteins of other viruses have been shown to interfere with the host antiviral response [60,61,62,63].

Recent studies on packaging of RVFV vRNAs showed that infected cells can produce empty and incomplete virions [19]. Specifically, about 40% of Clone13 virions produced on Vero cells completely lacked viral genomic RNA, and only about 10% of virions included all three genomic segments necessary for establishing an infection [19]. As shown by the creation of two- and four-segmented RVFV and by the rescue of the virus with the codon-shuffled M segment, the packaging of RVFV vRNA is adaptable and perhaps stochastic [17,18,19]. However, the presence of N is necessary for any RNAs to be packaged to nascent viral particles. Although direct interactions between RVFV glycoprotein and vRNAs are possible [12], it is expected that packaged RNAs are largely associated with N. Therefore, N interactions with host RNAs at least partially explain the production of empty and incomplete virions during RVFV infection.

### 4.2. N Binding Bias on vRNAs

Based on the cooperative binding model of N and RNA [25], we expected that vRNAs, especially viral genomic RNAs, would be completely coated by the N oligomer. The CLIP-seq coverage on vRNAs, however, showed uneven binding density of N to the vRNA species (Figure 4A). Whether N targeted specific sequence or structure motifs for binding is not yet clear. Moreover, we were surprised that we observed higher coverage on viral antisense RNAs than on viral sense RNAs in all segments (Figure 4B). Strikingly, the RT-qPCR analysis of the M segment (Figure 4C) showed that while there was equal expression of sense and antisense RNAs, the antisense species was more highly occupied by N, suggesting that the antisense M segment may possess more elements targeted by N for binding, or for steric reasons, N can more easily oligomerize along the RNA. It is also plausible that the requirement for intensive transcription of mRNAs from the genomic segments makes them less amenable to high static occupancy by N. It is also notable that the high N occupancy of the agM segment corresponds to efficient production of genomic M segments (Figure 4D–F, Supplemental Figure S4), suggesting that high coverage with N could contribute to efficient synthesis of the genomic segment, thereby highlighting potential mechanistic differences between mRNA transcription and generation of full-length replication intermediates.

### 4.3. Dynamics of N Expression and N-vRNA Interactions during RVFV Infection

To understand the dynamics of N protein expression, we developed a highly sensitive, quantitative proteomics method to accurately quantify N in RVFV-infected cells. The results showed that N expression first increased exponentially to reach a plateau around 10 hpi (Figure 5B). After the first exponential phase, N expression was increased only modestly even with higher MOI (Figure 5C). It was previously reported that, as a cellular response to RVFV infection, translational arrest occurs in the same time window as we observed the leveling of the N protein expression [46,64]. Thus, it may be critical for the virus that the rapid increase in intracellular N concentration occurs before the translational arrest to ensure successful progression of infection.

In contrast to the intracellular N protein concentration, which stayed relatively level after 10 hpi, vRNA expression steadily increased throughout the infection (Figure 4C). With rising vRNA expression, the N protein switches its preferred binding target from host RNAs to vRNAs (Figure 1B). As reported previously, intracellular abundance and the balanced expression of vRNAs are important for vRNA packaging [21,65]. The N–RNA interactions dictated by the concentration of both species may be a critical determinant for initiating a productive packaging process. Although our data suggest preferential N–vRNA interactions as genome levels rise, the specific sequence or structural motifs preferred by N and host RNAs remains to be elucidated. The published RVFV N structures were solved by co-crystalizing nonviral RNAs with N [24], thus some biologically relevant N–RNA interactions that are required during viral replication may not be represented. It may also be possible that, as shown in Crimean-Congo hemorrhagic fever virus, N is capable of two binding modes; one is specific for a sequence/structure motif and the other for nonspecific binding [66].

### 4.4. Formation and Roles of Incomplete Viral Particles

**Figure 7:**
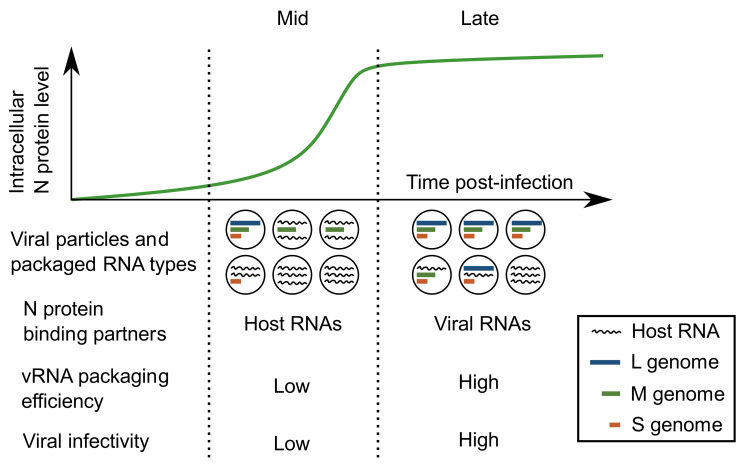
Formation of infectious and noninfectious viral particles. During the middle stage of infection (Mid), when N protein expression is actively increasing, the viral particles released include more host RNAs than vRNAs, resulting in low specific infectivity. At later stages of infection (Late), when N protein expression is stabilized and viral RNAs are at their peak abundance, the viral particles released include more vRNAs and have higher specific infectivity.

In this study, we examined the N-RNA interactions and viral particle production in RVFV-infected cells as a function of accumulation of vRNA species and N (Figure 7). We quantified the vRNA contents as well as fully infectious particles in cell culture media, which showed that the specific infectivity of the virus increases with infection time (Figure 6). In recent years, the formation of non-membrane-bound RNA-protein complexes has been gaining attention as a strategy to efficiently condense vRNA-protein complexes for packaging [67,68]. Formation of RNA-protein condensates is tightly controlled by the concentrations of both RNA and protein species [69]. For RVFV, the formation of supramolecular complexes that include all three genomic segments is not a prerequisite to viral genome packaging [19]. Though the formation of multi-segment, supramolecular complexes may not be important in RVFV infection, single-segment condensates may be used to produce viral particles that carry a subset of vRNA segments.

Viral particles that carry less than the full set of genomic segments are obviously not fully infectious by themselves. However, they can be useful for subsequent infection in neighboring cells. For example, assuming they package RdRp and one or more viral genome segment, incomplete particles could initiate a pre-infection with strategic expression of viral proteins. Subsequent collective infection with a complete or complementary virus particle could be more efficient and have a higher chance of producing more infectious virus. Although the prospect requires further investigation, (pre-)priming of neighboring cells with critical viral structural or host evasion proteins could be an attractive strategy for segmented viruses to successfully establish an infection.

Viral particles can disperse as a single particle or as an aggregated group [31]. If a particle containing the L and M segment traveled together with a particle containing the S segment only, the outcome of the group entering a cell would resemble a full infection caused by a complete virus. Layers of cells in natural hosts, such as epithelial cell layers that are known to be targeted by RVFV [70], are in close contact with each other. This would accentuate the virion aggregation that is a characteristic of cell-to-cell spread [31]. Relying on collective infection may be a simple strategy for viruses with a short one-step replication time [19,21].

These results suggest that there is not strong evolutionary selective pressure for RVFV to have a tightly controlled packaging mechanism, as dispersal of incomplete particles and collective infection of mammalian cells appear to be sufficient for robust virus spread between cells. Interestingly, RVFV packaging efficiency is different between mammalian and mosquito cells [21]. It would be of interest to precisely characterize the relationships between the N protein accumulation and the specific infectivity with multiple strains and in different hosts.

## Data Availability

All relevant MEM-MS data are presented in the manuscript. CLIP-seq data have been deposited in GEO database (GSE186067).

## Supplementary Materials

The following are available online at https://www.mdpi.com/article/10.3390/v13122417/s1, Figure S1: Stranded RT-qPCR primer design, Figure S2: Appending the constant region eliminates the primer-independent cDNA synthesis, Table S1: PCR primers used to prepare RT-qPCR standards, Table S2: RT and qPCR primers used in this study, Figure S3: RT-qPCR standard curves and statistics, Figure S4: RT-qPCR quantitation of vRNAs purified from MP-12-infected HEK293 cells.

## References

[1] Jupp P.G., Kemp A., Grobbelaar A., Leman P., Burt F.J., Alahmed A.M., Al Mujalli D., Al Khamees M., Swanepoel R. (2002). The 2000 epidemic of Rift Valley fever in Saudi Arabia: Mosquito vector studies. Med. Vet. Entomol..

[2] Bouloy M., Weber F. (2010). Molecular biology of rift valley Fever virus. Open Virol. J..

[3] Linthicum K.J., Britch S.C., Anyamba A. (2016). Rift Valley Fever: An Emerging Mosquito-Borne Disease. Annu. Rev. Entomol..

[4] Wilson M.L., Chapman L.E., Hall D.B., Dykstra E.A., Ba K., Zeller H.G., Traore-Lamizana M., Hervy J.P., Linthicum K.J., Peters C.J. (1994). Rift Valley fever in rural northern Senegal: Human risk factors and potential vectors. Am. J. Trop. Med. Hyg..

[5] McMillen C.M., Arora N., Boyles D.A., Albe J.R., Kujawa M.R., Bonadio J.F., Coyne C.B., Hartman A.L. (2018). Rift Valley fever virus induces fetal demise in Sprague-Dawley rats through direct placental infection. Sci. Adv..

[6] Baudin M., Jumaa A.M., Jomma H.J.E., Karsany M.S., Bucht G., Näslund J., Ahlm C., Evander M., Mohamed N. (2016). Association of Rift Valley fever virus infection with miscarriage in Sudanese women: A cross-sectional study. Lancet Glob. Health.

[7] Ikegami T., Makino S. (2011). The pathogenesis of rift valley fever. Viruses.

[8] Billecocq A., Spiegel M., Vialat P., Kohl A., Weber F., Bouloy M., Haller O. (2004). NSs Protein of Rift Valley Fever Virus Blocks Interferon Production by Inhibiting Host Gene Transcription. J. Virol..

[9] Bouloy M., Janzen C., Vialat P., Khun H., Pavlovic J., Huerre M., Haller O. (2001). Genetic Evidence for an Interferon-Antagonistic Function of Rift Valley Fever Virus Nonstructural Protein NSs. J. Virol..

[10] Overby A.K., Pettersson R.F., Neve E.P.A. (2007). The Glycoprotein Cytoplasmic Tail of Uukuniemi Virus (Bunyaviridae) Interacts with Ribonucleoproteins and Is Critical for Genome Packaging. J. Virol..

[11] Piper M.E., Sorenson D.R., Gerrard S.R. (2011). Efficient cellular release of Rift Valley fever virus requires genomic RNA. PLoS ONE.

[12] Tercero B., Narayanan K., Terasaki K., Makino S. (2021). Characterization of the Molecular Interactions That Govern the Packaging of Viral RNA Segments into Rift Valley Fever Phlebovirus Particles. J. Virol..

[13] Albarino C.G., Bird B.H., Nichol S.T. (2007). A shared transcription termination signal on negative and ambisense RNA genome segments of Rift Valley fever, sandfly fever Sicilian, and Toscana viruses. J. Virol..

[14] Ikegami T., Won S., Peters C.J., Makino S. (2007). Characterization of Rift Valley fever virus transcriptional terminations. J. Virol..

[15] Obijeski J.F., Bishop D.H., Palmer E.L., Murphy F.A. (1976). Segmented genome and nucleocapsid of La Crosse virus. J. Virol..

[16] Pettersson R.F., Von Bonsdorff C.H. (1975). Ribonucleoproteins of Uukuniemi virus are circular. J. Virol..

[17] Brennan B., Welch S.R., McLees A., Elliott R.M. (2011). Creation of a recombinant Rift Valley fever virus with a two-segmented genome. J. Virol..

[18] Wichgers Schreur P.J., Oreshkova N., Moormann R.J., Kortekaas J. (2014). Creation of Rift Valley fever viruses with four-segmented genomes reveals flexibility in bunyavirus genome packaging. J. Virol..

[19] Wichgers Schreur P.J., Kortekaas J. (2016). Single-Molecule FISH Reveals Non-selective Packaging of Rift Valley Fever Virus Genome Segments. PLoS Pathog..

[20] Terasaki K., Murakami S., Lokugamage K.G., Makino S. (2011). Mechanism of tripartite RNA genome packaging in Rift Valley fever virus. Proc. Natl. Acad. Sci. USA.

[21] Bermudez-Mendez E., Katrukha E.A., Spruit C.M., Kortekaas J., Wichgers Schreur P.J. (2021). Visualizing the ribonucleoprotein content of single bunyavirus virions reveals more efficient genome packaging in the arthropod host. Commun. Biol..

[22] Ferron F., Li Z., Danek E.I., Luo D., Wong Y., Coutard B., Lantez V., Charrel R., Canard B., Walz T. (2011). The hexamer structure of the Rift Valley fever virus nucleoprotein suggests a mechanism for its assembly into ribonucleoprotein complexes. PLoS Pathog..

[23] Raymond D.D., Piper M.E., Gerrard S.R., Smith J.L. (2010). Structure of the Rift Valley fever virus nucleocapsid protein reveals another architecture for RNA encapsidation. Proc. Natl. Acad. Sci. USA.

[24] Raymond D.D., Piper M.E., Gerrard S.R., Skiniotis G., Smith J.L. (2012). Phleboviruses encapsidate their genomes by sequestering RNA bases. Proc. Natl. Acad. Sci. USA.

[25] Hornak K.E., Lanchy J.-m., Lodmell J.S. (2016). RNA Encapsidation and Packaging in the Phleboviruses. Viruses.

[26] Overby A.K., Popov V.L., Pettersson R.F., Neve E.P. (2007). The cytoplasmic tails of Uukuniemi Virus (Bunyaviridae) G(N) and G(C) glycoproteins are important for intracellular targeting and the budding of virus-like particles. J. Virol..

[27] Ellenbecker M., Sears L., Li P., Lanchy J.M., Stephen Lodmell J. (2012). Characterization of RNA aptamers directed against the nucleocapsid protein of Rift Valley fever virus. Antivir. Res..

[28] Ellenbecker M., Lanchy J.M., Lodmell J.S. (2014). Inhibition of Rift Valley fever virus replication and perturbation of nucleocapsid-RNA interactions by suramin. Antimicrob Agents Chemother.

[29] Jiao L., Ouyang S., Liang M., Niu F., Shaw N., Wu W., Ding W., Jin C., Peng Y., Zhu Y. (2013). Structure of Severe Fever with Thrombocytopenia Syndrome Virus Nucleocapsid Protein in Complex with Suramin Reveals Therapeutic Potential. J. Virol..

[30] Carnec X., Ermonval M., Kreher F., Flamand M., Bouloy M. (2014). Role of the cytosolic tails of Rift Valley fever virus envelope glycoproteins in viral morphogenesis. Virology.

[31] Sanjuán R., Thoulouze M.I. (2019). Why viruses sometimes disperse in groups?. Virus Evol..

[32] Martin M. (2011). Cutadapt removes adaptor sequences from high-throughput sequencing reads. EMBnet. J..

[33] Dobin A., Davis C.A., Schlesinger F., Drenkow J., Zaleski C., Jha S., Batut P., Chaisson M., Gingeras T.R. (2013). STAR: Ultrafast universal RNA-seq aligner. Bioinformatics.

[34] Kondili M., Fust A., Preussner J., Kuenne C., Braun T., Looso M. (2017). UROPA: A tool for Universal Robust Peak Annotation. Sci. Rep..

[35] Robinson J.T., Thorvaldsdóttir H., Winckler W., Guttman M., Lander E.S., Getz G., Mesirov J.P. (2011). Integrative genomics viewer. Nat. Biotechnol..

[36] Afgan E., Baker D., Batut B., van den Beek M., Bouvier D., Cech M., Chilton J., Clements D., Coraor N., Grüning B.A. (2018). The Galaxy platform for accessible, reproducible and collaborative biomedical analyses: 2018 update. Nucleic. Acids Res..

[37] Shihavuddin A., Basu S., Rexhepaj E., Delestro F., Menezes N., Sigoillot S.M., Del Nery E., Selimi F., Spassky N., Genovesio A. (2017). Smooth 2D manifold extraction from 3D image stack. Nat. Commun..

[38] Holden P., Horton W.A. (2009). Crude subcellular fractionation of cultured mammalian cell lines. BMC Res. Notes.

[39] Vashist S., Urena L., Goodfellow I. (2012). Development of a strand specific real-time RT-qPCR assay for the detection and quantitation of murine norovirus RNA. J. Virol. Methods.

[40] Chen C., Ridzon D.A., Broomer A.J., Zhou Z., Lee D.H., Nguyen J.T., Barbisin M., Xu N.L., Mahuvakar V.R., Andersen M.R. (2005). Real-time quantification of microRNAs by stem-loop RT-PCR. Nucleic. Acids Res..

[41] Wenger C.D., Coon J.J. (2013). A proteomics search algorithm specifically designed for high-resolution tandem mass spectra. J. Proteome Res..

[42] Moore M.J., Zhang C., Gantman E.C., Mele A., Darnell J.C., Darnell R.B. (2016). Erratum: Mapping Argonaute and conventional RNA-binding protein interactions with RNA at single-nucleotide resolution using HITS-CLIP and CIMS analysis. Nat. Protoc..

[43] Wheeler E.C., Van Nostrand E.L., Yeo G.W. (2018). Advances and challenges in the detection of transcriptome-wide protein-RNA interactions. Wiley Interdiscip Rev. RNA.

[44] Havranek K.E., White L.A., Lanchy J.M., Lodmell J.S. (2019). Transcriptome profiling in Rift Valley fever virus infected cells reveals modified transcriptional and alternative splicing programs. PLoS ONE.

[45] Copeland A.M., Altamura L.A., Van Deusen N.M., Schmaljohn C.S. (2013). Nuclear relocalization of polyadenylate binding protein during rift valley fever virus infection involves expression of the NSs gene. J. Virol..

[46] Hopkins K.C., Tartell M.A., Herrmann C., Hackett B.A., Taschuk F., Panda D., Menghani S.V., Sabin L.R., Cherry S. (2015). Virus-induced translational arrest through 4EBP1/2-dependent decay of 5’-TOP mRNAs restricts viral infection. Proc. Natl. Acad. Sci. USA.

[47] Ruigrok R.W., Crepin T., Kolakofsky D. (2011). Nucleoproteins and nucleocapsids of negative-strand RNA viruses. Curr. Opin. Microbiol..

[48] Ikegami T., Won S., Peters C.J., Makino S. (2006). Rescue of infectious rift valley fever virus entirely from cDNA, analysis of virus lacking the NSs gene, and expression of a foreign gene. J. Virol..

[49] Murakami S., Terasaki K., Ramirez S.I., Morrill J.C., Makino S. (2014). Development of a novel, single-cycle replicable rift valley Fever vaccine. PLoS Negl. Trop. Dis..

[50] Ahsan N.A., Sampey G.C., Lepene B., Akpamagbo Y., Barclay R.A., Iordanskiy S., Hakami R.M., Kashanchi F. (2016). Presence of Viral RNA and Proteins in Exosomes from Cellular Clones Resistant to Rift Valley Fever Virus Infection. Front. Microbiol..

[51] Timani K.A., Liao Q., Ye L., Zeng Y., Liu J., Zheng Y., Yang X., Lingbao K., Gao J., Zhu Y. (2005). Nuclear/nucleolar localization properties of C-terminal nucleocapsid protein of SARS coronavirus. Virus Res..

[52] Wulan W.N., Heydet D., Walker E.J., Gahan M.E., Ghildyal R. (2015). Nucleocytoplasmic transport of nucleocapsid proteins of enveloped RNA viruses. Front. Microbiol..

[53] Wang R., Brattain M.G. (2007). The maximal size of protein to diffuse through the nuclear pore is larger than 60 kDa. FEBS Lett..

[54] Han J., Xiong J., Wang D., Fu X.D. (2011). Pre-mRNA splicing: Where and when in the nucleus. Trends Cell Biol..

[55] Barnes B., Lubyova B., Pitha P.M. (2002). On the role of IRF in host defense. J. Interferon Cytokine Res. Off. J. Int. Soc. Interferon Cytokine Res..

[56] Zhou A., Dong X., Liu M., Tang B. (2021). Comprehensive Transcriptomic Analysis Identifies Novel Antiviral Factors Against Influenza A Virus Infection. Front. Immunol..

[57] Perwitasari O., Cho H., Diamond M.S., Gale M. (2011). Inhibitor of κB kinase epsilon (IKK(epsilon)), STAT1, and IFIT2 proteins define novel innate immune effector pathway against West Nile virus infection. J. Biol. Chem..

[58] Lin R.J., Yu H.P., Chang B.L., Tang W.C., Liao C.L., Lin Y.L. (2009). Distinct antiviral roles for human 2^′^,5^′^;-oligoadenylate synthetase family members against dengue virus infection. J. Immunol..

[59] Bréhin A.C., Casadémont I., Frenkiel M.P., Julier C., Sakuntabhai A., Desprès P. (2009). The large form of human 2^′^,5^′^-Oligoadenylate Synthetase (OAS3) exerts antiviral effect against Chikungunya virus. Virology.

[60] Liu Y., Liang Q.Z., Lu W., Yang Y.L., Chen R., Huang Y.W., Wang B. (2021). A Comparative Analysis of Coronavirus Nucleocapsid (N) Proteins Reveals the SADS-CoV N Protein Antagonizes IFN-β Production by Inducing Ubiquitination of RIG-I. Front. Immunol..

[61] Lu X., Pan J., Tao J., Guo D. (2011). SARS-CoV nucleocapsid protein antagonizes IFN-β response by targeting initial step of IFN-β induction pathway, and its C-terminal region is critical for the antagonism. Virus Genes.

[62] Carnec X., Baize S., Reynard S., Diancourt L., Caro V., Tordo N., Bouloy M. (2011). Lassa virus nucleoprotein mutants generated by reverse genetics induce a robust type I interferon response in human dendritic cells and macrophages. J. Virol..

[63] Ontiveros S.J., Li Q., Jonsson C.B. (2010). Modulation of apoptosis and immune signaling pathways by the Hantaan virus nucleocapsid protein. Virology.

[64] Brennan B., Welch S.R., Elliott R.M. (2014). The consequences of reconfiguring the ambisense S genome segment of Rift Valley fever virus on viral replication in mammalian and mosquito cells and for genome packaging. PLoS Pathog..

[65] Murakami S., Terasaki K., Narayanan K., Makino S. (2012). Roles of the coding and noncoding regions of rift valley Fever virus RNA genome segments in viral RNA packaging. J. Virol..

[66] Jeeva S., Mir S., Velasquez A., Ragan J., Leka A., Wu S., Sevarany A.T., Royster A.D., Almeida N.A., Chan F. (2019). Crimean-Congo hemorrhagic fever virus nucleocapsid protein harbors distinct RNA-binding sites in the stalk and head domains. J. Biol. Chem..

[67] Cubuk J., Alston J.J., Incicco J.J., Singh S., Stuchell-Brereton M.D., Ward M.D., Zimmerman M.I., Vithani N., Griffith D., Wagoner J.A. (2021). The SARS-CoV-2 nucleocapsid protein is dynamic, disordered, and phase separates with RNA. Nat. Commun..

[68] Brocca S., Grandori R., Longhi S., Uversky V. (2020). Liquid-Liquid Phase Separation by Intrinsically Disordered Protein Regions of Viruses: Roles in Viral Life Cycle and Control of Virus-Host Interactions. Int. J. Mol. Sci..

[69] Banerjee P.R., Milin A.N., Moosa M.M., Onuchic P.L., Deniz A.A. (2017). Reentrant Phase Transition Drives Dynamic Substructure Formation in Ribonucleoprotein Droplets. Angew. Chem. Int. Ed. Engl..

[70] Oymans J., Wichgers Schreur P.J., van Keulen L., Kant J., Kortekaas J. (2020). Rift Valley fever virus targets the maternal-foetal interface in ovine and human placentas. PLoS Negl. Trop. Dis..

