# Supplementary material for: Time-Resolved Analysis of N-RNA Interactions during RVFV Infection Shows Qualitative and Quantitative Shifts in RNA Encapsidation and Packaging
Source: Viruses. 2021 Dec 2;13(12):2417. doi: 10.3390/v13122417 (PMC8704896; doi:10.3390/v13122417)
Supplement: Supplementary file 1 [file viruses-13-02417-s001.zip › viruses-1443007-supplementary.pdf]

Title

Time-resolved analysis of N-RNA interactions during RVFV infection shows qualitative and quantitative shifts in RNA encapsidation and packaging

Miyuki Hayashi, Eric P. Schultz, Jean-Marc Lanchy, and J. Stephen Lodmell

Supplementary Figures and Tables

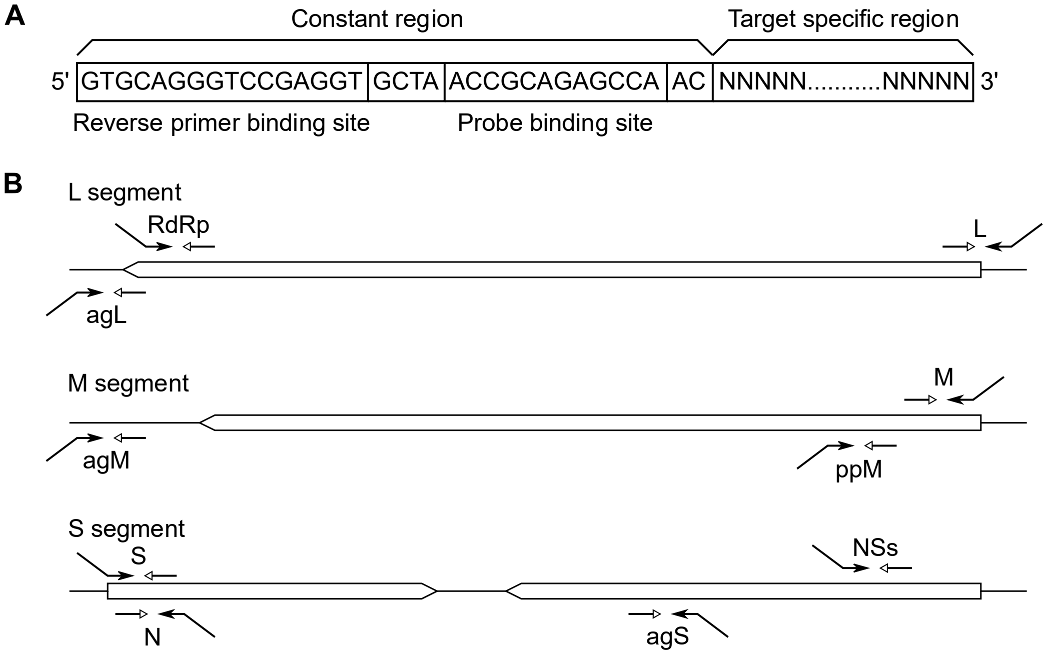

Figure S1: Stranded RT-qPCR primer design. (A) The design of the reverse transcription (RT) primer used in this study. Appending a constant region to an RT primer is shown to eliminate the production of primer-independent complementary DNA (cDNA) products that may cause over estimation of the target transcript in the subsequent qPCR step [1]. During the preparation of this manuscript, the similar method has been developed and used by Tercero et al. [2,3]. The constant region was designed based on the sequence used by Chen et al. [4]. (B) The regions targeted by each primer.

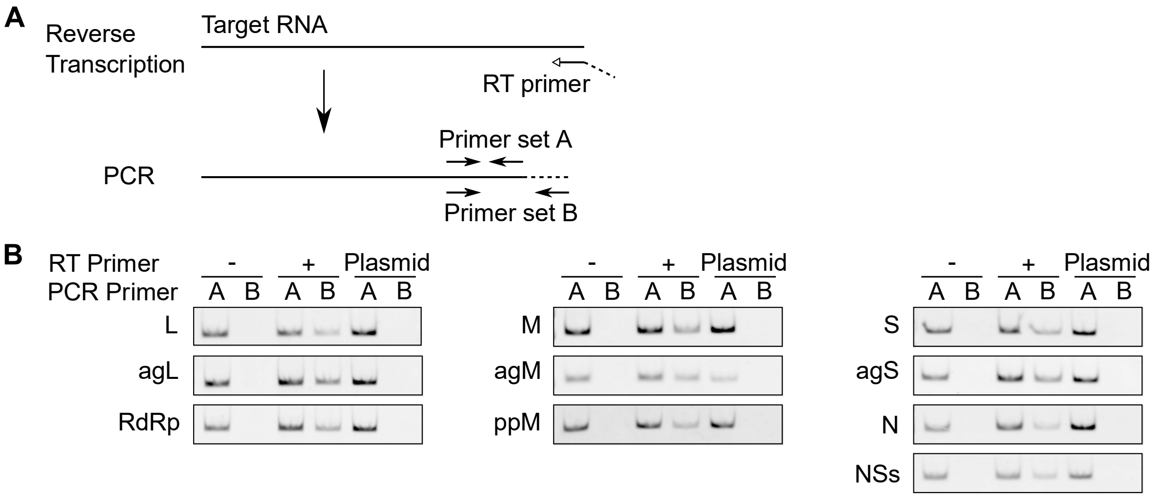

Figure S2: Appending the constant region eliminates the primer-independent cDNA synthesis. (A) The experimental set up. Target viral RNAs were purified from RVFV MP-12 infected HEK293 cells. After RT reaction, the end-point PCR experiment was conducted by using two different primer sets. Both forward and reverse primers in Primer set A anneals to the viral RNA sequence, whereas the reverse primer in Primer set B anneals to the constant region of the RT primer (shown as a broken line). Primer set B is used in RT-qPCR experiments shown in this study. (B) Products of the end-point PCR reactions are visualized on non-denaturing acrylamide gels. Primer set A produced signal in absence of RT primer. Primer set B signal arises only when the strand-specific RT primer is added to the reaction.

Table S1: PCR primers used to prepare RT-qPCR standards. Target sequences were amplified from pTVT7-GS, -GM, and -GL plasmid by using the primers listed below. The bolded letters indicate the sequence recognized by T7 polymerase. L = genomic L segment, agL = antigenomic L segment, RdRp = RNA-dependent RNA polymerase, M = genomic M segment, agM = antigenomic M segment, ppM = polyprotein M, S = genomic S segment, agS = antigenomic S segment, NSs = nonstructural protein S, N = nucleocapsid protein.

Target	Name	Sequence	Note
L	T7-L_F	**TAATACGACTCACTATAGGG**GGCAGCTGAATAGTTGACTTGTGG	-
L	L_R	ACACAAAGGCGCCCAATCA	-
agL	T7-agL_F	**TAATACGACTCACTATAGGG**GGATGCATTCATTCAGGATGCAAG	-
agL	agL_R	ACACAAAGACCGCCCAATATTG	-
RdRp	RdRp_R	CCACATGGATTCCAATACTAGC	Used with T7-agL_F
M	T7-M_F	**TAATACGACTCACTATAGGG**TGCTGAGTTGGCCATCACAC	-
M	M_R	ACACAAAGACGGTGCATTAAATGT	-
agM	T7-agM_F	**TAATACGACTCACTATAGGG**GCCTTAGGGCACCAAACCT	-
agM	agM_R	ACACAAAGACCGGTGCAACT	-
ppM	ppM_T7_R	TGCAAAGGGCACAACCTCAT	Used with T7-agM_F
S	T7-S_F	**TAATACGACTCACTATAGGG**ACACAAAGACCCCCTAGTGC	-
S	S_R	ACACAAAGCTCCCTAGAGATACAA	-
NSs	NSs_R	GGCAGCCTTAACCTCTAATCAACC	Used with T7-S_F
agS	T7-agS_F	**TAATACGACTCACTATAGGG**ACACAAAGCTCCCTAGAGATACAA	-
agS	agS_R	ACACAAAGACCCCCTAGTGC	-
N	N_R	CTAATCCCGACCGTAACCCC	Used with T7-agS_F

Table S2: RT and qPCR primers used in this study. Bolded letters indicate the constant region on the RT primer. [+C] indicates locked-nucleic acid base. The abbreviations used in the 'Target' column is the same as what's been used in Table 1.

Target	Name	Sequence
L	L_RT	**GTGCAGGGTCCGAGGTGCTATCCGCAGAGCCAAC**GCTAACTCTGGCACTTCCAAC
L	L_F	GAGCCTATTTCAGATGCTCCTTGT
agL	agL_RT	**GTGCAGGGTCCGAGGTGCTATCCGCAGAGCCAAC**ACACAAAGACCGCCCAATATTGT
agL	agL_F	GACCAGTAAGCAAAGTCAGGC
RdRp	RdRp_RT	**GTGCAGGGTCCGAGGTGCTATCCGCAGAGCCAAC**TCCGCCACATCTGTCTCC
RdRp	RdRPp_F	CGGTGCTCCAGCAAAGACTA
M	M_RT	**GTGCAGGGTCCGAGGTGCTATCCGCAGAGCCAAC**GGTGACTCCACTAACCCAGAG
M	M_F	GAGGTCTTAACCTCTCTTATGCCTG
agM	agM_RT	**GTGCAGGGTCCGAGGTGCTATCCGCAGAGCCAAC**ACACAAAGACCGGTGCAACT
agM	agM_F	GCAGCAGTCTCAAGTGCTTG
ppM	ppM_RT	**GTGCAGGGTCCGAGGTGCTATCCGCAGAGCCAAC**AGTGGAGTCACCAAAGCAGG
ppM	ppM_F	TCGGTTCTGGTGTGTGAAGC
S	S_RT	**GTGCAGGGTCCGAGGTGCTATCCGCAGAGCCAAC**CTTGCGATCCAGTTTGCTGC
S	S_F	AAGCAAACTCTCGGACCCAC
agS	agS_RT	**GTGCAGGGTCCGAGGTGCTATCCGCAGAGCCAAC**AGGTTGCTCACGTACAGTGC
agS	agS_F	CAGCATCAGGCTCTCCTCC
N	N_RT	**GTGCAGGGTCCGAGGTGCTATCCGCAGAGCCAAC**AACTCTACGGGCATCAAACC
N	N_F	ATCAAGAGCTTGCGATCCAG
NSs	NSs_RT	**GTGCAGGGTCCGAGGTGCTATCCGCAGAGCCAAC**CACAACAGGGCCCAACCATA
NSs	NSs_F	CAGAGTGGTCGTCGTGTTGT
-	RVFV_probe	FAM-TGG[+C]T[+C]TG[+C]GGA-BHQ1
-	Universal_R	GTGCAGGGTCCGAGGT

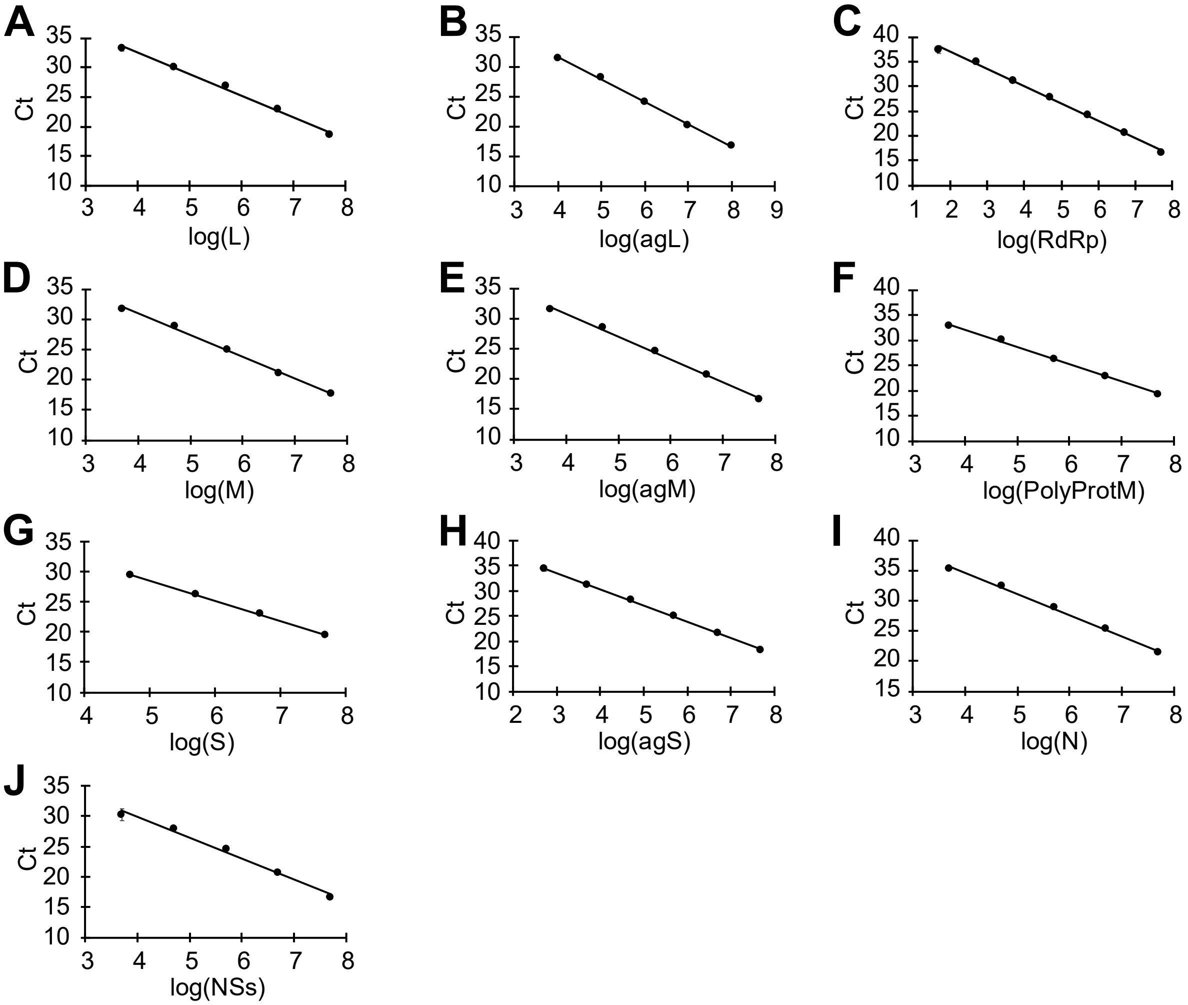

K

Target	Slope	y-Intercept	R^2	Efficiency (%)
L	-3.66	47.17	0.994	87.74
agL	-3.73	46.47	0.998	97.92
RdRp	-3.51	44.10	0.997	92.59
M	-3.61	45.50	0.997	89.17
agM	-3.77	47.11	0.997	84.06
PolyProtM	-3.30	42.60	0.986	100.72
S	-3.31	45.07	0.999	100.43
agS	-3.22	43.22	0.999	104.33
N	-3.50	48.68	0.997	93.04
NSs	-3.45	43.67	0.990	94.83

Figure S3: RT-qPCR standard curves and statistics. (A-J) Standard curves generated from dilutions of in vitro transcribed viral RNAs. The observed Ct values are plotted against raw copy numbers of input RNA. (K) Slope, y-intercept, R^2, and qPCR efficiency of each primer set.

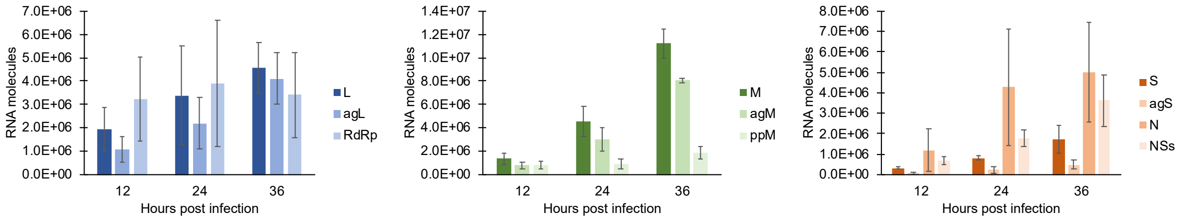

Figure S4: RT-qPCR quantitation of vRNAs purified from MP-12 infected HEK293 cells. Means and SEMs of two repeated experiments at cell passage numbers 2 and 6 are plotted. Each experiment had 2 or more biological replicates.

References

1. Vashist, S.; Urena, L.; Goodfellow, I. Development of a strand specific real-time RT-qPCR assay for the detection and quantitation of murine norovirus RNA. *J Virol Methods* **2012**, *184*, 69-76, doi:10.1016/j.jviromet.2012.05.012.
2. Tercero, B.; Terasaki, K.; Nakagawa, K.; Narayanan, K.; Makino, S. A strand-specific real-time quantitative RT-PCR assay for distinguishing the genomic and antigenomic RNAs of Rift Valley fever phlebovirus. *J Virol Methods* **2019**, *272*, 113701, doi:10.1016/j.jviromet.2019.113701.
3. Tercero, B.; Narayanan, K.; Terasaki, K.; Makino, S. Characterization of the Molecular Interactions That Govern the Packaging of Viral RNA Segments into Rift Valley Fever Phlebovirus Particles. *J Virol* **2021**, *95*, e0042921, doi:10.1128/JVI.00429-21.
4. Chen, C.; Ridzon, D.A.; Broomer, A.J.; Zhou, Z.; Lee, D.H.; Nguyen, J.T.; Barbisin, M.; Xu, N.L.; Mahuvakar, V.R.; Andersen, M.R.; et al. Real-time quantification of microRNAs by stem-loop RT-PCR. *Nucleic Acids Research* **2005**, *33*, doi:10.1093/nar/gni178.
